# A Novel Stoichio-Kinetic Model for the DPPH• Assay: The Importance of the Side Reaction and Application to Complex Mixtures

**DOI:** 10.3390/antiox10071019

**Published:** 2021-06-24

**Authors:** Lucrezia Angeli, Sebastian Imperiale, Yubin Ding, Matteo Scampicchio, Ksenia Morozova

**Affiliations:** Faculty of Science and Technology, Free University of Bozen-Bolzano, Piazza Università 1, 39100 Bolzano, Italy; lucrezia.angeli@natec.unibz.it (L.A.); sebastian.imperiale@unibz.it (S.I.); yubin.ding@natec.unibz.it (Y.D.); ksenia.morozova@unibz.it (K.M.)

**Keywords:** DPPH assay, kinetic model, phenolic compounds, side reaction, high-resolution mass spectrometry, antioxidant activity

## Abstract

The 2,2-diphenyl-1-picrylhydrazyl (DPPH•) assay is widely used to determine the antioxidant activity of food products and extracts. However, the common DPPH• protocol uses a two-point measurement and does not give information about the kinetics of the reaction. A novel stoichio-kinetic model applied in this study monitors the consumption of DPPH• by common antioxidants following the second order reaction. The fitting of such decay yields the rate constant k_1_, which describes the main reaction between antioxidants and DPPH•, and the rate constant k_2_, which is attributed to a slower side reaction considering the products generated between the transient radicals (AO•) and another molecule of DPPH•. The model was first applied to antioxidant standards. Sinapic acid, Trolox and ascorbic and chlorogenic acids did not show any side reaction. Instead gallic, ferulic and caffeic acids achieved the best fitting with k_2_. The products of the side reaction for these compounds were confirmed and identified with high-resolution mass spectrometry. Finally, the kinetic model was applied to evaluate the antioxidant activity of eight herbal extracts. This study suggests a new kinetic approach to standardize the common DPPH• assay for the determination of antioxidant activity.

## 1. Introduction

2,2-Diphenyl-1-picrylhydrazyl (DPPH•) is a stable free radical that is commonly used to characterize the antioxidant activity of methanolic extracts in the so-called DPPH• assay. DPPH• is widely used to assess the antioxidant activity of food extracts thanks to the velocity of the reaction, the facility in the measurement and the stability of the radical.

The utility of the DPPH• assay to measure the antioxidant activity of herbal extracts has been debated [1]. The common protocol is based on the single-point measurement of the absorbance of DPPH• at its maximum (usually 515 nm) after 30 min or 1 h reaction. A limitation of the common assays is that they fail to provide temporal information that can be used to distinguish the rate at which different antioxidants produce their antioxidant effect [2]. Many works express results reporting the EC_50_ value, or as percentage of inhibition or Trolox or ascorbic acid equivalents [3,4]. However, this measurement is a single time-point and provides different results in the literature due to the solvent effect [5,6,7,8]. Indeed, the rate constant decreases in aprotic solvents and increases in alcohols, demonstrating that a main electron-transfer mechanism is involved rather than H-atom transfer [9]. Several new and alternative approaches have been revised in recent years, underlining the drawbacks of the lack of a standardized method [10,11,12,13,14,15].

Kinetic approaches to analyzing DPPH• data have been recently proposed [2,16,17,18,19,20]. They are considered the only useful parameters to predict the antioxidant ability of an extract [1]. The mechanism involved in the reaction of the DPPH radical is described in Equation (1):

(1) $$\mathrm{AOH}+{\mathrm{DPPH}}^{*}\overset{k_{1}}{\to }{\mathrm{AO}}^{*}+\mathrm{DPPH}-H$$

However, the possible side reactions occurring between the transient radicals (AO•) generated in the first reaction with DPPH• have not been fully considered yet. Such radicals are generally able to quench another DPPH radical, as illustrated in Equation (2) [21].

(2) $${\mathrm{AO}}^{*}+{\mathrm{DPPH}}^{*}\overset{k_{2}}{\to }\;\mathrm{products}$$

This reaction is largely underestimated and could explain the variability of the results reported in the literature [22,23,24,25]. Recently, Foti and colleagues accounted for this side reaction. They measured the reactivity of antioxidants with the DPPH radical applying a kinetic model of pseudo-first (when antioxidants are in excess) or second order (when the concentration of DPPH• is equal to or higher than that of antioxidants) [9]. The authors were able to deduce the presence of side reactions graphically by following the DPPH• decay over time. However, proof of this side reaction based on a mechanistic model has not been demonstrated yet.

Thus, the aim of this work was to develop a mechanistic kinetic model that could be used to fit experimental data of the reaction between antioxidant compounds and DPPH• by a numerical integration method. Using this approach, not only can the kinetic rate constant of the primary reactions between antioxidants and DPPH• be achieved, but the rate constants of the slower side reaction and the stoichiometric factors can also be easily determined. Accordingly, the kinetic reaction of DPPH• and different concentrations of common antioxidants, including Trolox and ascorbic, gallic, ferulic, caffeic, sinapic and chlorogenic acids was analyzed using this kinetic approach. Validation of the proposed mechanism of the reaction and the detection of oxidation products has also been achieved using UHPLC-MS/MS data [26,27,28]. Finally, this approach was applied to measure the antioxidant activity of several herbal extracts. Undertaking this type of study is important to determine a consolidated methodology to assess the antioxidant activity of simple and complex mixtures. The side reaction could enhance the performance of the DPPH• assay by minimizing the errors resulting from not considering this kinetic mechanism. Furthermore, it could bring new understanding of how the antioxidant activity should be expressed.

## 2. Materials and Methods

### 2.1. Chemicals

The 2,2-dyphenyl-1-picrylhydrazyl (DPPH•—C_18_H_12_N_5_O_6_) with a purity higher than 98%, trans-ferulic acid (C_10_H_10_O_4_), gallic acid (C_7_H_6_O_5_), chlorogenic acid (C_16_H_18_O_9_), caffeic acid (C_9_H_8_O_4_), sinapic acid (C_11_H_12_O_5_), Trolox, ethanol with a purity higher than 99.8% and sodium carbonate with a purity higher than 99% were all purchased from Sigma-Aldrich (St. Louis, MO, USA). LC-MS grade methanol, and LC-MS grade acetonitrile were both purchased from Honeywell (Muskegon, MI, USA), and Folin–Ciocalteu’s reagent was purchased from Titolchimica (Pontecchio polesine, Italy).

### 2.2. Herbal Sample Extraction

Herbal samples (10 g) of *Moringa oleifera*, *Turnera aphrodisiaca*, *Urtica dioica*, *Rhodiola rosea*, *Melissa officinalis*, *Fraxinus excelsior* and *Filipendula ulmaria* were ground (PerkinElmer, Laboratory Mill 3100, Waltham, MA, USA) and extracted with 100 mL of Milli-Q/EtOH 1:1 *v*/*v* facilitated by ultrasound equipment (STEEL^®^, digital ultrasonic generator, 575 W, Unitech, Padua, Italy) at 60% power for 40 min at 35 °C. After extraction, the extracts were filtered with 0.45 µM PTFE filters (Whatman^TM^, Maidstone, UK) and centrifuged (SL 16R Centrifuge, Thermo Scientific, Waltham, MA, USA) at 20 °C at 10,000 rpm for 10 min to remove all herbal residues. The extracts were stored at −80 °C until further use. The extracts were diluted 150 times and were subjected to Folin–Ciocalteu’s method to obtain a µM concentration of gallic acid equivalents (GAEs). After that, the concentration expressed as GAE was standardized so that the final concentration of the extracts was not higher than ¼ of the DPPH• concentration.

### 2.3. Standard Solution Preparation

Stock solutions (10 mM) in methanol were prepared for caffeic acid, trans-ferulic acid, chlorogenic acid, gallic acid, sinapic acid, ascorbic acid and Trolox. Several dilutions were made to reach different concentrations (0.05, 0.1, 0.25, 0.5, 1, 2 and 4 mM). A 1.25 mM stock solution of DPPH radical was prepared daily in methanol. All stock solutions were prepared the same day as the analyses, sonicated for 3 min and filtered with 0.45 µm PTFE filters (Whatman^TM^, Maidstone, UK).

### 2.4. Stoichio-Kinetic Modelling of DPPH• Assay

The decay of DPPH• was measured at its maximum absorbance at 515 nm with a Cary 60 UV-VIS spectrophotometer (Agilent Technology, Santa Clara, CA, USA). A 100 µM DPPH• solution in methanol usually had maximum absorbance at 1.1 ± 0.4 (ε in methanol = 10,870 ± 200 M^−1^cm^−1^) [9]. The following procedure was used to determine the value of the rate constants and stoichiometric factor (n) for all phenols and extracts. First, 800 µL of a 125 µM DPPH• working solution (final concentration 100 µM) was transferred into a quartz cuvette, while 200 µL of the antioxidant solutions was injected with a syringe to prevent the loss of data during the initial seconds of the reaction. Different concentrations of antioxidants (10, 20, 50, 100, 200, 400 and 800 µM) reacted with 100 µM DPPH•, and the absorbance decay was measured from the beginning to 3 min for gallic and ascorbic acid; to 5 min for caffeic acid, Trolox and sinapic acid; to 10 min for chlorogenic acid and to 15 min for ferulic acid. The concentration of the DPPH• was deduced from the Beer–Lambert law, and a concentration versus time graph was generated. To calculate the rate constants and the stoichiometry of the reaction, different models were created in the software Copasi, and the best fitting is reported in Section 3.1 (see below).

### 2.5. Analysis of Products of Reaction by High Resolution Mass Spectrometry

The reaction between the standards and DPPH• was performed at a molar ratio of ca. 1:3 AOH:DPPH•, where AOH is the antioxidant. The mixture was analyzed after 1 h reaction at 25 °C and avoiding contact with light. A blank (methanol) was performed before, between every injection and at the end of the sequence. The products formed after the reaction of antioxidants with the DPPH• radical were analyzed using a Dionex Ultimate 3000 UHPLC device (Thermo Fisher Scientific, Waltham, MA, USA) coupled to a Q Exactive^TM^ Orbitrap high resolution mass spectrometer (Thermo Fisher Scientific, Waltham, MA, USA). For analysis, the method of Berton et al. [27] was applied with some modifications. Briefly, the separation was carried out by using a Resteck ROC C18 5 µm column (150 × 4.6 mm) at a flow rate of 1 mL/min at 40 °C. The gradient mixture of solvents A (Milli-Q water) and B (acetonitrile) was set as follows: 5% (*v*/*v*) B (0–1 min), from 5 to 30% B (1–3 min), from 30 to 92% B (3–14 min), and up to 95% B (14–18 min), hold at 95% (18–22 min), down to 5% B (22–25 min) and hold at 5% B (25–27 min). For full MS analysis, the electrospray source was operated in a negative ionization mode with a capillary voltage of 4.5 kV at a temperature of 350 °C. The scan range was set at 135–1500 *m*/*z* with an acquisition rate of 1 microscan per second and a resolution of 70,000 (at 200 *m*/*z*), AGC target at 2 × 10^5^ and maximum injection time of 100 ms. The parameters of the data dependent MS^2^ acquisition were as follows: dd-MS^2^ AGC target 1 × 10^5^, maximum injection time 50 ms, resolution 17,500, loop count 5, isolation window 4.0 *m*/*z*, isolation offset 1 *m*/*z* and normalized collision energy 30 eV. A different method was used for gallic acid products. The gradient mixture of solvents A (0.1% *v*/*v* formic acid in Milli-Q water) and B (methanol) was set as follows: 5% B (0–3 min), 30% B (3–12 min), 92% B (12–13 min), 95% B (13–17 min), 5% B (17–20 min). For full MS analysis, the electrospray source was operated in a negative ionization mode with a capillary voltage of 3.2 kV at a temperature of 320 °C. The scan range was set at 135–1500 *m*/*z* with an acquisition rate of 1 microscan per second and a resolution of 35,000 (at 200 *m*/*z*), AGC target at 5 × 10^5^ and maximum injection time of 65 ms. The parameters of the data dependent MS^2^ acquisition were as follows: dd-MS^2^ AGC target 5 × 10^5^, maximum injection time 75 ms, resolution 17,500 and loop count 15. The parameters not mentioned were set as described above.

The final non-radical products yielded from the reaction between each antioxidant and DPPH• were tentatively identified through appropriate molecular formulas, using the extracted ion chromatogram, and confirmed by the fragmentation spectra.

### 2.6. Absorbance Scan

The absorbance scan from 200 to 700 nm was registered for gallic acid/DPPH• 10:100 µM after 2 min reaction.

### 2.7. Folin–Ciocalteu Total Phenol Content

To assess the molar concentration of the herbal extracts expressed as µM of gallic acid equivalent (GAE), 13 µL of each extract previously diluted 145 times was mixed for 20 s with 100 µL of Milli-Q water and 13 µL of Folin–Ciocalteu’s reagent in a well plate. After 5 min, 13 µL of a saturated sodium carbonate solution was manually added to each well, and absorbance was recorded at 765 nm after 2 h reaction. The GAEs were calculated through a gallic acid calibration curve (*R*^2^ = 0.9995), and every sample was measured in triplicate.

### 2.8. Statistics

Copasi calculated all the parameters through the best-fitting equation in a differential evolution program that minimizes the error between the experimental data and the fitted values. The two rate constants were calculated from a two-system equation that also accounts for the n value [29]. For other calculations, such as mean and standard deviation, Microsoft Excel was used. A one-way ANOVA with Tukey’s post-hoc test was performed using the program XLSTAT, considering a *p*-value of 0.05.

## 3. Results and Discussion

### 3.1. Stoichio-Kinetic Model for the DPPH• Assay

#### 3.1.1. The Simple Model of Sinapic Acid

**Figure 1:**
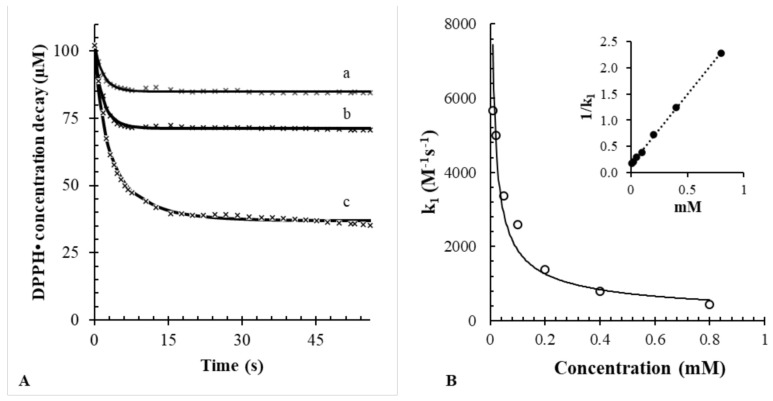
(**A**) Best fitting model for sinapic acid at different concentrations vs. 100 µM DPPH• employing Equation (1). a: 10; b: 20; c: 50 µM. (**B**) Observed rate constants versus concentration; the solid line represents the potential equation. The inset represents the linear trend of concentrations vs. 1/k_1_ (*R*^2^ = 0.999).

Figure 1A shows the effect of sinapic acid on the decay of DPPH• (bullet points). The bimolecular reaction was studied at 25 °C, after the rapid mixing of DPPH• into a cuvette containing sinapic acid dissolved in methanol. The transient changes of DPPH• were continuously monitored by measuring the absorbance signal at 515 nm (ε in methanol = 10,870 ± 200 M^−1^ cm^−1^). In all the experiments, the initial concentration of DPPH• was 100 µM. The initial concentration of sinapic acid was varied from 10 to 800 µM.

Figure 1B also shows the best fitting (solid lines) that matched the experimental points (white circles). The fitting was performed with nonlinear regression by minimizing the gap between the experimental and predicted concentrations of DPPH•. Typically, given the reaction mechanism (see Equation (1)), the initial concentration of DPPH• (100 µM) and the initial concentrations of products (all equal to zero), the iterative nonlinear least-squares fitting routine finds the best estimates of the rate constant k_1_ and the predicted concentration of sinapic acid. Overall, the results confirm that the reaction between DPPH• and sinapic acid follows a second order rate law (*R*^2^ = 0.999). The high degree of fitting may exclude the presence of significant side reactions for sinapic acid.

The stoichiometric factor n was also calculated according to Equation (3), as the ratio between the initial sinapic acid concentration estimated using the iterative fitting routine and the effective concentration of sinapic acid used in the experiment:

(3) $$n=\frac{{\left[\mathrm{sinapic}\right]}_{\mathrm{Copasi}}}{{\left[\mathrm{sinapic}\right]}_{\mathrm{known}}}$$

For example, if the iterative fitting routine predicts an initial concentration of antioxidant of 20 µM, but the real concentration used in the cuvette is 10 μM, then the stoichiometric factor is n = 2. Alternatively, the stoichiometric factor could be directly derived graphically from Figure 1 as the ratio between the DPPH• concentration loss and the initial concentration of sinapic acid. For example, if the initial concentration of antioxidant is 10 µM, but the loss of DPPH• corresponds to 20 μM, then the stoichiometric factor, again, is equal to n = 2. Both approaches indicated that the stoichiometric factor of sinapic acid greatly depends on the DPPH• to sinapic acid ratio. In details, when DPPH• was in excess (2–5 times larger than that the concentration of sinapic acid), the stoichiometric factor was nearly equal to 2 (see Table 1). When the ratio AOH:DPPH• increased to 1 or higher, the stoichiometric factor dropped significantly. This is because when sinapic acid is in excess with respect to DPPH•, the reaction becomes limited by the concentration of radicals. This result highlights the importance of always performing the DPPH• assay in excess of the DPPH• reagent [30].

A further result is that the rate constant k_1_ varies as a function of AOH following a nonlinear trend (Figure 1B). The inset of Figure 1B shows that the reciprocal of k_1_ is linearly related to the concentration of sinapic acid (*R*^2^ = 0.999). A similar effect of AOH on k_1_ for other antioxidants was reported previously [9].

As for sinapic acid, other common antioxidants such as Trolox and ascorbic acid show a similar kinetic behavior (see Table 2). These antioxidants have very high rate constants and a stoichiometric factor close to 2. This is consistent with a mechanism involving the rapid transfer of two electrons and two protons [31,32].

#### 3.1.2. A More Complex Model: The Introduction of the Side Reaction

The mechanism of Equation (1) cannot be applied to all antioxidants. In some cases, as with gallic acid, this simple mechanism is unable to accurately predict the change of the DPPH• concentration over time. In addition, the stoichiometric value for gallic acid (n = 5) is unrealistic because it cannot be explained using its chemical structure [33]. Indeed, gallic acid has three hydroxyl groups involved in antioxidant activity, not five. However, accurate results can instead be achieved if the mechanism described in Equation (1) is followed by a further reaction (Equation (2)), which is referred to as a “side reaction”. This secondary reaction step is not widely used in the literature, although it was earlier proposed by Foti et al. [9]. It provides a satisfactory description of the progress of more complex reaction kinetics, such as those between DPPH• and gallic acid.

**Figure 2:**
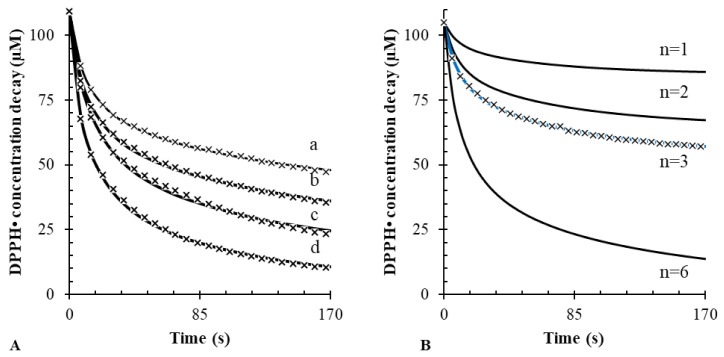
(**A**) Best fitting considering both Equations (1) and (2) at different concentrations of gallic acid (a: 10; b: 20; c: 50; d: 100 µM) vs. 100 µM DPPH•; (**B**) the precision of the software while calculating n is reported. Only for n = 3 was best fitting obtained.

The need to account for the side reaction is clearly highlighted in Figure 2A. This experiment shows the experimental DPPH• decay obtained upon the addition of varying concentrations of gallic acid. Also shown is the corresponding fitting line obtained by nonlinear regression with the reaction mechanism that includes Equations (1) and (2). When both reactions are considered, the resulting coefficient of determination is much higher (*R*^2^ > 0.999) than that obtained with the model without the side reaction (*R*^2^ < 0.988). Furthermore, as depicted in Figure 2B, the stoichiometry of the reaction can be also unequivocally identified.

The same iterative fitting routine was then applied to determine the rate constants, k_1_ and k_2_, of several antioxidants. Results are reported in Table 2. Overall, from the comparison of the results obtained with or without the side reaction, it is evident that the side reaction improves the fitting of the kinetic model. Even more importantly, the side reaction can be used to obtain stoichiometric factors that are predictable from the chemical structure of the antioxidants. When the side reaction was not included in the model, the resulting k_1_ was underestimated.

#### 3.1.3. Validation of the Model by Analysis of Reaction Products

**Figure 3:**
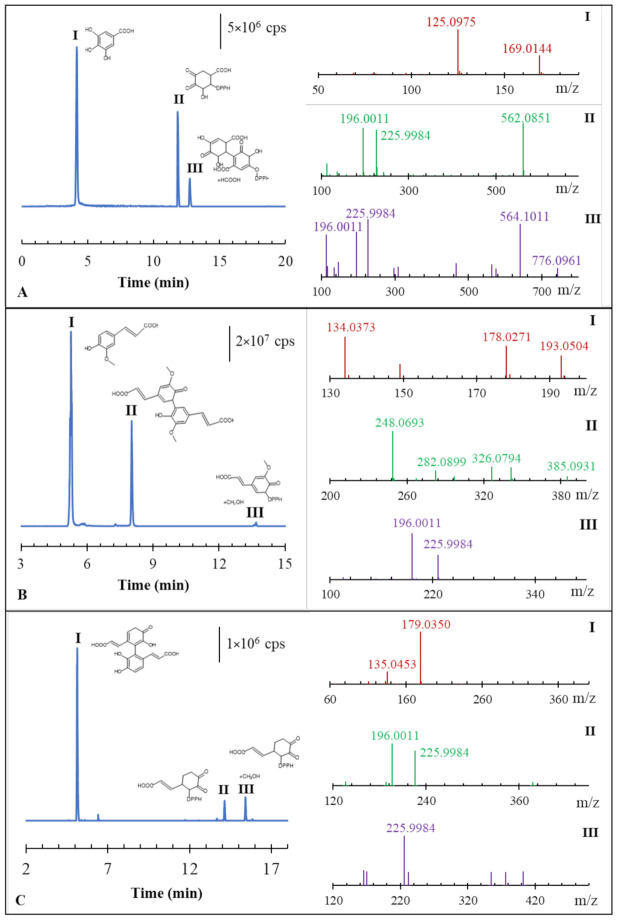
Extracted ion chromatographic peaks with the corresponding proposed molecular formulas for gallic (**A**), ferulic (**B**) and caffeic acid (**C**) after the reaction with DPPH•. On the right, the dd-MS^2^ spectra presenting the characteristic fragment ions are reported.

The presence of a side reaction, and thus the validity of Equation (2), was next investigated using liquid chromatography coupled with high resolution mass spectrometry (LC-MS). For such experiments, antioxidants 1–7 were left to react with DPPH• at a 1:3 ratio. Then, an aliquot was injected into the LC system, and the main products of the reactions were identified by MS full scan, followed by a data dependent acquisition of the resulting MS/MS spectra. Typical results for selected antioxidants are plotted in Figure 3. Overall, the analysis of the reaction products revealed two distinctive behaviors among the antioxidants selected for this study.

The first facile behavior was observed for antioxidants such as ascorbic acid, Trolox, sinapic acid and chlorogenic acid. When such antioxidants were left to react with DPPH•, the main reaction products observed (see Supplementary Materials Figures S1–S4), were the stable quinonic form or, in the case of sinapic and chlorogenic acid, a dimer. The lack of side reactions is consistent with the previous results of the DPPH• kinetic assay (see Table 1 and Table 2), where k_2_ was negligible. Only for chlorogenic acid was a small side reaction identified. The finding of no reaction products can be justified by the very low value of k_2_ proposed by the kinetic model. Indeed, the *R*^2^ value was also very good (*R*^2^ > 0.999) in the model without the side reaction. When ascorbic acid reacted with DPPH•, the main observed mass fragments were those of dehydroascorbic acid (i.e., the oxidized form of ascorbic acid, with a molecular ion of *m*/*z* 173.0089), which was found at a retention time (RT) of 1.63 min. Analogously, the main mass fragments for the reaction products of Trolox were those of the oxidized form, with a *m*/*z* value of 265.1081 (RT = 7.18 min). However, in the case of sinapic acid and chlorogenic acid, the presence of a dimeric form was also noticed, with mass fragments of *m*/*z* 445.1141 (RT = 7.99 min) and *m*/*z* 735.1773 (RT = 4.80 min), respectively. This m/z value represents the dimer with the addition of methanol. However, such dimers were not connected with the side reaction mechanism of DPPH•. Indeed, traces of the same dimer were also found during the analysis of sinapic acid standard. This supports the hypothesis that the occurrence of such dimers could be attributed to a self-oxidation reaction of the antioxidant.

The second type of behavior was observed for the group of gallic, caffeic and ferulic acid. With such antioxidants, the semi-quinonic form produced with Equation (1) was followed by a further reaction with DPPH•, consistent with Equation (2) (i.e., the side reaction). This secondary reaction generated a new stable, non-radical complex, composed of the antioxidant (i.e., gallic, caffeic or ferulic acid) and DPPH•. For instance, when gallic acid reacted with DPPH•, three chromatographic peaks were recorded (see Figure 3A). The first peak (“I”) corresponded to the gallic acid monomer, with a molecular ion of *m*/*z* 169.0144. The second peak (“II”) corresponded to the complex between DPPH• and the partial oxidized gallic acid radical, with a molecular ion of *m*/*z* 562.0851. The presence of DPPH• in such complex products was demonstrated by dd-MS^2^ analysis, which revealed fragments typical of the ionization products of DPPH• at *m*/*z* 225.9984 and 196.0011. The same two ions were also observed in control experiments with DPPH• alone (i.e., without gallic acid), together with the molecular ion of DPPH• at *m*/*z* 394.0794. Finally, the third peak (“III”) was characterized by a molecular ion at *m*/*z* 776.0961. This larger mass ion was identified as a complex adduct between DPPH• and the gallic acid dimer plus formic acid (which was present in the eluent phase). The dd-MS2 spectra of this fragment again revealed the typical fragment ions of DPPH• at *m*/*z* 196.0011 and 225.9984. Overall, these findings provide evidence of the formation of products from the side reaction between DPPH• and gallic acid. This also confirmed our previous findings with the DPPH• kinetic method and the need to also include Equation (2) in the kinetic model.

**Figure 4:**
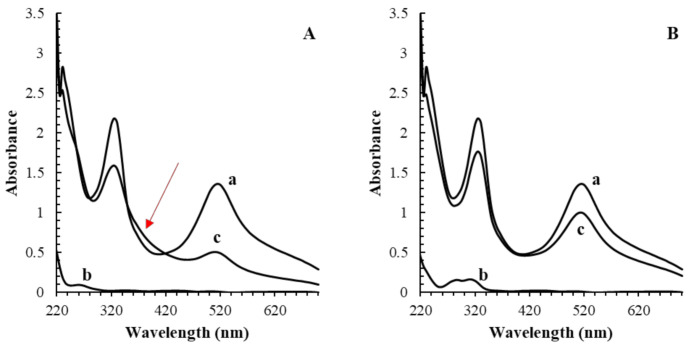
Comparison of the absorbance scans of 100 µM DPPH• (a), 10 µM antioxidant (b) and the mixture (c) after 2 min reaction in gallic (**A**) and ferulic acid (**B**). The atypical absorbance trend for the reaction of gallic acid and DPPH• between 350 and 400 nm was explained by the presence of potential reaction products.

Further confirmation of the existence of the side reaction between gallic acid and DPPH• was also confirmed by recording the absorbance spectrum from 200 to 700 nm; as illustrated in Figure 4A, an atypical absorbance wave at about 350–400 nm was registered after 2 min of the reaction between gallic acid and DPPH• (ratio 1:10). Indeed, many compounds, among them hexahydroxydiphenic acid, have a maximum of absorbance at ca. 360 nm [34].

The presence of the side reaction was also observed for the case of ferulic acid (Figure 3B). Indeed, peak “III” of the three chromatographic peaks reported corresponds to the adduct of ferulic acid with DPPH• and methanol with a molecular ion at *m*/*z* 619.1552 (RT = 13.68 min). The dd-MS^2^ spectra of this fragment revealed the presence of DPPH• by showing the typical fragment ions. Peak “I” represents ferulic acid, which was eluted after 5.33 min with a molecular ion at *m*/*z* 193.0504. Other typical fragments at *m*/*z* 134.0373 and 176.0271 characterized the peak “I”. However, the analysis also revealed the dimer of ferulic acid with a molecular ion at *m*/*z* 385.0931 (RT = 8.08 min) [27]. As highlighted above, the presence of the dimer is not involved in the pathway of the side reaction.

Finally, Figure 3C shows three chromatographic peaks for the reaction products of caffeic acid with DPPH•. In this case, the side reaction is also confirmed by peak “II” and “III”. Peak II was eluted after 14.05 and peak III after 15.31 min, with the corresponding molecular ions at *m*/*z* 575.1295 and 606.1477. Both the peaks show the typical fragments of DPPH• in the dd-MS^2^ spectra, and they were not found in the analysis of the caffeic acid standard alone or DPPH• alone. They correspond to the product of caffeic acid and DPPH•, respectively, one in the pure form and the other one with the addition of methanol (CH_3_OH). Peak “I” corresponds to the dimer for caffeic acid, which was eluted after 5.09 min with a molecular ion of *m*/*z* 357.0615. In this case, the dimer is not implied in the side reaction mechanism.

Overall, these findings supported the presence of side reactions between antioxidants such as gallic, ferulic and caffeic acid [27,35,36].

However, the presence of side reactions was validated, as shown in Figure 3, with the other antioxidants, such as gallic acid, ferulic acid, caffeic acid and chlorogenic acid, confirming the results in Table 2. All molecular formulas and theoretical and experimental masses are reported in Table 3.

### 3.2. Application of the Model to Herbal Extracts: Prediction of the Kinetics

**Figure 5:**
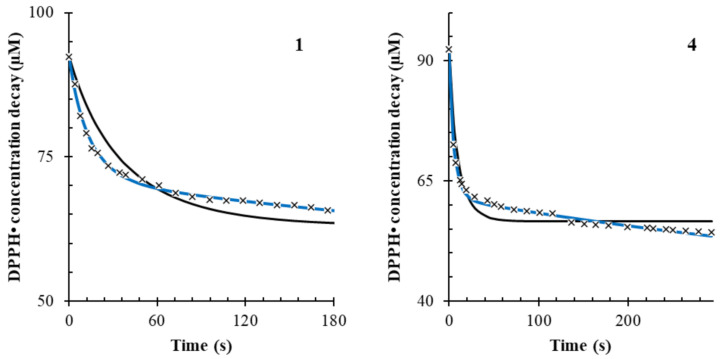
Fitting of the kinetic data for *Moringa oleifera* (**1**) and *Harpagophytum procubens* (**4**). The black solid line represents the fitting in the model without the side reaction, and the blue line represents the fitting in the model with the side reaction. The “x” points represent the data points for the recorded concentration decay of 100 µM DPPH•.

Finally, the stoichio-kinetic model described was applied to evaluate the antioxidant activity of eight herbal extracts, in particular *Moringa oleifera* (1), *Filipendula ulmaria* (2), *Fraxinus excelsior* (3), *Harpagophytum procubens* (4), *Melissa officinalis* (5), *Rhodiola rosea* (6), *Turnera aphrodisiaca* (7) and *Urtica dioica* (8), by registering the decay in absorbance at 515 nm of 100 µM DPPH•. The Folin–Ciocalteu method was applied to the extracts to express their total phenolic content as gallic acid equivalents (GAE). Because of the need to always run the kinetic DPPH• assay under an excess of DPPH• reagent in respect to the antioxidant, all the extracts were diluted to obtain nearly the same equivalent concentration of gallic acid (ca. 25 µM GAE). Figure 5 shows the observed decay of the DPPH• concentration and the resulting best fitting obtained by iterative nonlinear regression of the kinetic model involving the side reaction (Equations (1) and (2)).

Accordingly, extracts 2, 4 and 8 showed the highest k_1_ value (*p* < 0.001), indicating a very fast scavenging mechanism (Equation (1)). Extracts 5, 6 and 7 showed the highest k_2_ values (*p* < 0.001), indicating the stronger presence of a side reaction mechanism compared to other herbs. Therefore, a higher value of k_2_ is related to a longer scavenging mechanism over time. The results are reported in Table 4. The stoichiometric factor, n’, is the ratio between the concentration obtained from Copasi and the estimation obtained from Folin–Ciocalteu’s method. Thus, such a measure is only apparent and should not be taken as an absolute value.

## 4. Conclusions

In this study, the stoichio-kinetic model succeeded in describing the complex reaction mechanism of the DPPH•. The presence of a side reaction was demonstrated. For several antioxidants, a kinetic study without considering the side reaction would cause the underestimation of the corresponding rate constant and the misleading estimation of the stoichiometry factor. Moreover, new products of the reaction were characterized for gallic acid and caffeic acid, providing new knowledge of the reaction pathway.

The significance of these achievements could lead to new studies that better characterize the reaction mechanism in complex matrices and elucidate why a kinetic model is superior to single-point measurement methods. The main limitation of the current study is that it is difficult to say which of the two reactions (main or side) is the most important for the antioxidant activity. Thus, further in vivo studies are needed to explain the role of the side reaction in relation to the effective ability of antioxidants to protect an oxidizable substrate. In particular, future work should explain if an antioxidant with a very high k_1_ value could protect better an oxidizable substrate than an antioxidant with lower k_1_ value but higher k_2_ value. Therefore, there is a need to assess whether the k_1_ or the k_2_ values should be considered when expressing the absolute power of an antioxidant. This would provide a standardized method to classify antioxidants.

## Figures and Tables

**Table 1:** Different concentrations of sinapic acid corresponding to different k_1_ values. The *R*^2^ value refers to the fitting between experimental values and fitted values. The stoichiometric factor, n, is calculated from the mean of the obtained n(i) of 10 and 20 µM.

Phenol	[AOH] (µM)	k_1_ (10^3^ M^−1^s^−1^)	SD	*R* ^2^	n(i)	n
Sinapic acid	10	5.67	0.33	0.997	1.8	1.7
	20	4.99	0.30	0.987	1.5	
	50	3.37	0.29	0.991	1.3	
	100	2.59	0.10	0.999	1.0	
	200	1.37	0.18	0.995	0.5	
	400	0.80	0.04	0.997	0.3	
	800	0.44	0.03	0.999	0.1	

**Table 2:** Observed rate constants, k_1_ and k_2_ (M^−1^s^−1^), *R*^2^ values and stoichiometric factors, n, for the reaction of DPPH• 100 µM with antioxidants (2–7) 10 and 100 µM at 25 °C in methanol, without and with side reaction. The values obtained are the mean of at least three repetitions, with a standard deviation of max 20%.

Antioxidants	Results without Side Reaction			Results with Side Reaction			
	k_1_ (10^3^ M^−1^s^−1^)	*R* ^2^	n	k_1_ (10^3^ M^−1^s^−1^)	k_2_ (M^−1^s^−1^)	*R* ^2^	n
2 gallic acid	0.42–0.57	0.987	4.9	1.13–0.66	145–23	0.997	2.9
3 caffeic acid	0.7–0.24	0.985	2.6	0.81–0.51	4–110	0.993	2.4
4 chlorogenic acid	0.19–0.04	0.999	2.3	0.2–0.04	0.6–8	0.999	2.1
5 ferulic acid	0.11–0.08	0.984	1.3	0.33–0.15	45–26	0.998	0.8
6 ascorbic acid	11.5–3.63	0.996	1.9	11.5–3.63	-	0.996	1.9
7 Trolox	0.54–0.39	0.999	2.2	0.54–0.39	-	0.999	2.2

**Table 3:** Proposed compounds with corresponding molecular formula and theoretical and experimental mass.

Proposed Compound	Molecular Formula	Theoretical Mass (*m*/*z*)	Experimental Mass (*m*/*z*)
DPPH•	C_18_H_12_N_5_O_6_	394.0793	394.0794
Gallic acid	C_7_H_6_O_5_	169.0143	169.0144
Adduct gallic + DPPH	C_25_H_17_N_5_O_11_	562.0852	562.0851
Adduct gallic dimer + DPPH + HCOOH	C_33_H_23_N_5_O_18_	776.0965	776.0961
Ferulic acid	C_10_H_10_O_4_	193.0506	193.0504
Ferulic dimer	C_20_H_18_O_8_	385.0929	385.0931
Adduct ferulic + DPPH + CH_3_OH	C_29_H_25_N_5_O_11_	619.1556	619.1552
Caffeic acid	C_9_H_8_O_4_	179.035	179.0349
Caffeic dimer	C_18_H_13_O_8_	357.0616	357.0615
Adduct caffeic + DPPH	C_27_H_21_N_5_O_10_	575.1294	575.1295
Adduct caffeic + DPPH + CH_3_OH	C_28_H_24_N_5_O_11_	606.1478	606.1477
Chlorogenic acid	C_16_H_18_O_9_	353.0878	353.0878
Chlorogenic quinonic form	C_16_H_16_O_9_	351.0722	351.0724
Chlorogenic dimer + CH_3_OH	C_33_H_36_O_19_	735.1778	735.1773
Sinapic acid	C_11_H_12_O_5_	223.0612	223.0612
Sinapic dimer	C_22_H_22_O_10_	445.114	445.1141
Ascorbic acid	C_6_H_8_O_6_	175.0248	175.0246
Dehydroascorbic acid	C_6_H_6_O_6_	173.0092	173.0089
Trolox	C_14_H_18_O_4_	249.1132	249.1134
Trolox oxidized	C_14_H_18_	265.1082	265.1081

**Table 4:** The values for k_1_ and k_2_ are the mean between at least three replicates. AH_0_ (Copasi) refers to the mean of the replicates of the M concentration estimated by Copasi, while AH_0_ (Folin) refers to the concentration (M GAE) obtained from Folin–Ciocalteu’s method. The apparent stoichiometric factor, n’, is the ratio between the estimated AH_0_ and the one measured by Folin–Ciocalteu’s method.

Extract	k_1_ (10^3^ M^−1^s^−1^)	SD	k_2_ (M^−1^s^−1^)	SD	AH_0_ (Copasi) (10^6^ M)	SD	AH_0_ (Folin) (10^6^ M)	n’	*R* ^2^
1	0.61	0.01	20.6	4.6	13.9	2.0	27.0	0.5	0.997
2	1.14	0.10	29.1	6.7	34.1	1.3	26.5	1.3	0.995
3	0.52	0.05	23.8	7.3	27.9	1.6	27.0	1.0	0.998
4	1.20	0.18	18.6	2.4	22.7	7.6	26.8	0.7	0.991
5	0.48	0.07	71.1	4.9	21.3	3.3	26.8	0.8	0.997
6	0.88	0.14	123	21	33.9	4.8	26.7	1.3	0.998
7	0.76	0.04	77.1	4.5	12.7	7.0	26.5	0.5	0.996
8	1.11	0.11	21.8	3.2	28.7	1.8	26.9	1.1	0.997

## Data Availability

The data published in this study are available on request from the corresponding author. The data are not publicly available due to privacy reasons.

## Supplementary Materials

The following are available online at https://www.mdpi.com/article/10.3390/antiox10071019/s1, Figure S1: chromatographic peaks and mass spectra for ascorbic acid products, Figure S2: chromatographic peaks and mass spectra for sinapic acid products, Figure S3: chromatographic peaks and mass spectra for Trolox products, Figure S4: chromatographic peaks and mass spectra for chlorogenic acid products.
