# Supplementary material for: A Novel Stoichio-Kinetic Model for the DPPH• Assay: The Importance of the Side Reaction and Application to Complex Mixtures
Source: Antioxidants (Basel). 2021 Jun 24;10(7):1019. doi: 10.3390/antiox10071019 (PMC8300744; doi:10.3390/antiox10071019)
Supplement: Supplementary file 1 [file antioxidants-10-01019-s001.zip › antioxidants-1261948-supplementary.pdf]

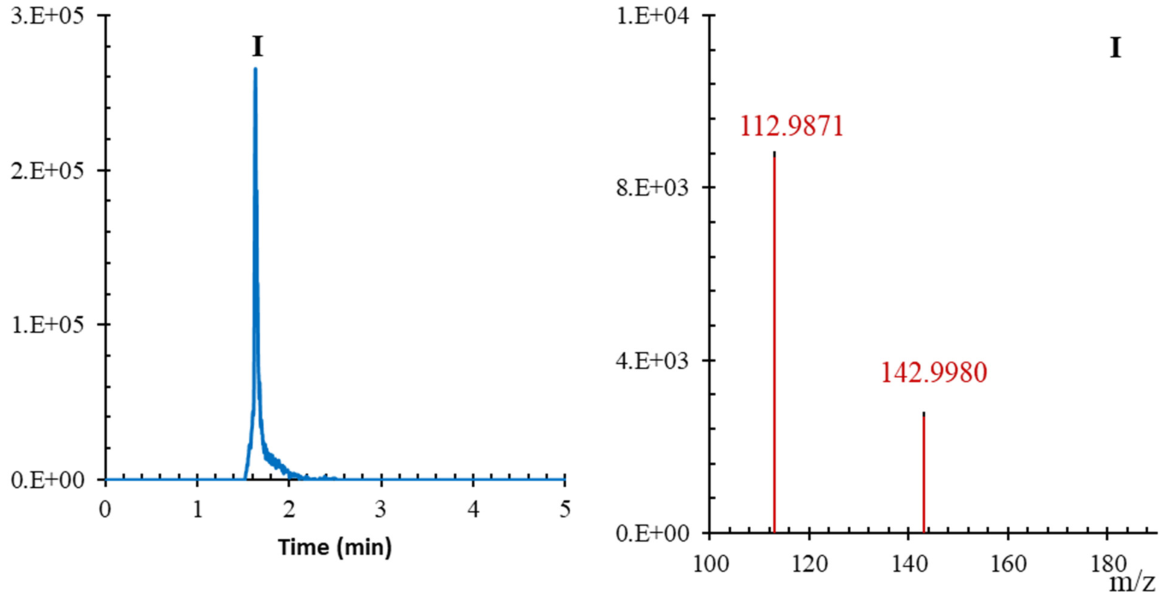

Figure S1. Chromatographic peak of dehydroascorbic acid and relative fragmentation spectrum.

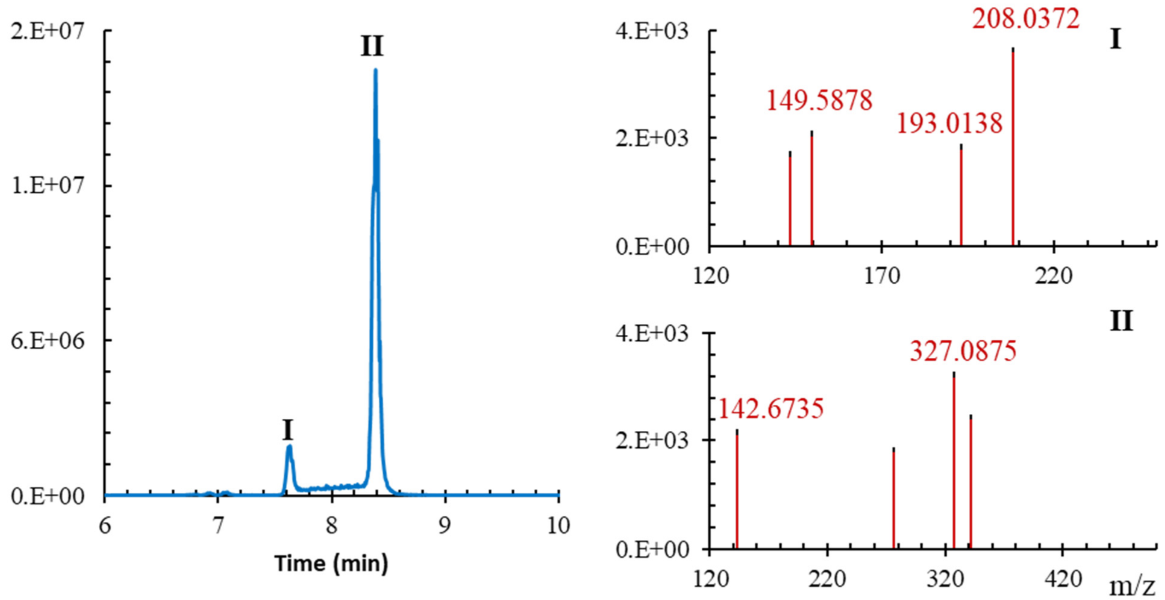

Figure S2. Chromatographic peaks of sinapic acid (I) and dimer of sinapic acid (II) and relative fragmentation spectra.

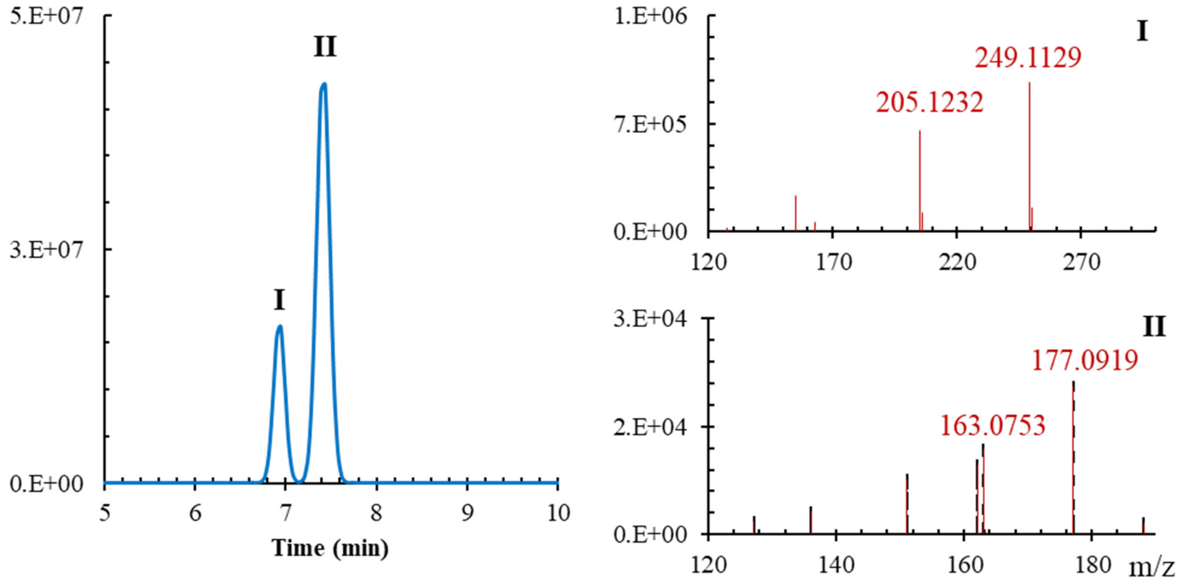

Figure S3. Chromatographic peaks of trolox (I) and Trolox oxidized (II) and relative fragmentation spectra.

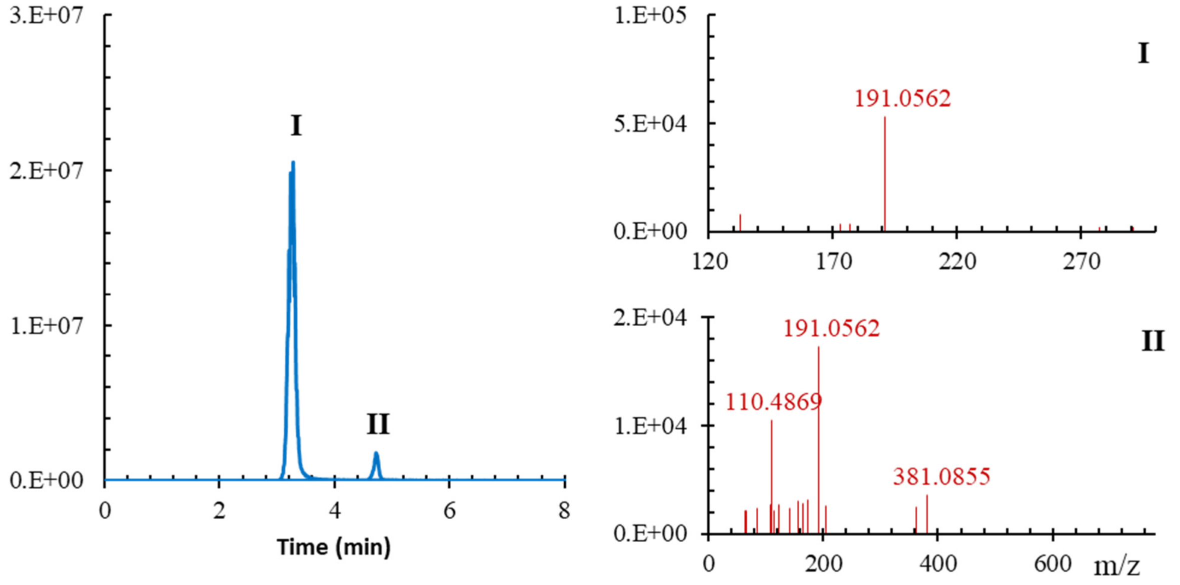

Figure S4. Chromatographic peaks of the oxidized form of chlorogenic acid (I) and the dimer (II) and relative fragmentation spectra.
